# A Case Report of Anesthesia-induced Diffuse Alveolar Hemorrhage Presenting to the Emergency Department

**DOI:** 10.5811/cpcem.1381

**Published:** 2023-10-18

**Authors:** Daniel Yiu, Christopher Stephens, Jacquelyn McCullough, Lesley Osborn

**Affiliations:** * The University of Texas Health Science Center at Houston, McGovern Medical School Emergency Medicine, Houston, Texas; † The University of Texas Health Science Center at Houston, Department of Anesthesiology, Houston, Texas; ‡ The University of Texas Health Science Center at Houston, McGovern Medical School, Houston, Texas; § The University of Texas Health Science Center at Houston, Department of Emergency Medicine, Houston, Texas

**Keywords:** diffuse alveolar hemorrhage, respiratory distress, anesthesia, critical care

## Abstract

**Introduction:**

The inhaled anesthetic sevoflurane is an uncommon etiology of diffuse alveolar hemorrhage (DAH). As DAH typically presents in the inpatient, postoperative setting, it has been infrequently reported in the anesthesiology literature and, to our knowledge, has not been reported in the emergency medicine literature to date.

**Case Report:**

We describe the presentation of a young, healthy male in respiratory distress to a busy urban emergency department (ED) after an outpatient surgical procedure. We highlight the etiology of post-anesthesia DAH and the acute management of this rare diagnosis in the ED.

**Conclusion:**

With outpatient surgical centers becoming an increasingly popular option for lower risk procedures, emergency physicians would benefit from understanding this presentation and its pathophysiology.

## INTRODUCTION

Diffuse alveolar hemorrhage (DAH) secondary to the inhaled, volatile anesthetic sevoflurane is an uncommon etiology with few case reports in the anesthesia literature and none in the emergency medicine (EM) literature to date. This readily available and frequently used general anesthetic has a long safety record with few significant side effects. Many patients undergoing general anesthesia receive sevoflurane in both inpatient and outpatient surgical centers.

## CASE REPORT

We report the case of a 20-year-old healthy male who was transported by emergency medical services (EMS) to the emergency department (ED) from an outpatient surgery center with a chief complaint of shortness of breath and hemoptysis. The patient reported that upon awakening from anesthesia in the post-anesthesia care unit (PACU), he was coughing up blood clots. He was in the PACU after undergoing general endotracheal anesthesia for an outpatient shoulder tendon repair procedure. Of note, the patient received inhaled sevoflurane for anesthesia and an inter-scalene nerve block for postoperative pain control. The patient reported no significant past medical history and no smoking or drug use history. Family history was significant for a postoperative deep venous thrombosis (DVT) after surgery in the patient’s mother and systemic lupus erythematous in his maternal aunt. The patient was of athletic build, participated in track and field, and had no prior history of receiving general anesthesia. Brief discussion with the attending anesthesiologist from the outpatient surgery center revealed no further details. He reported no difficulty with induction, intubation, or extubation.

**Image 1.**
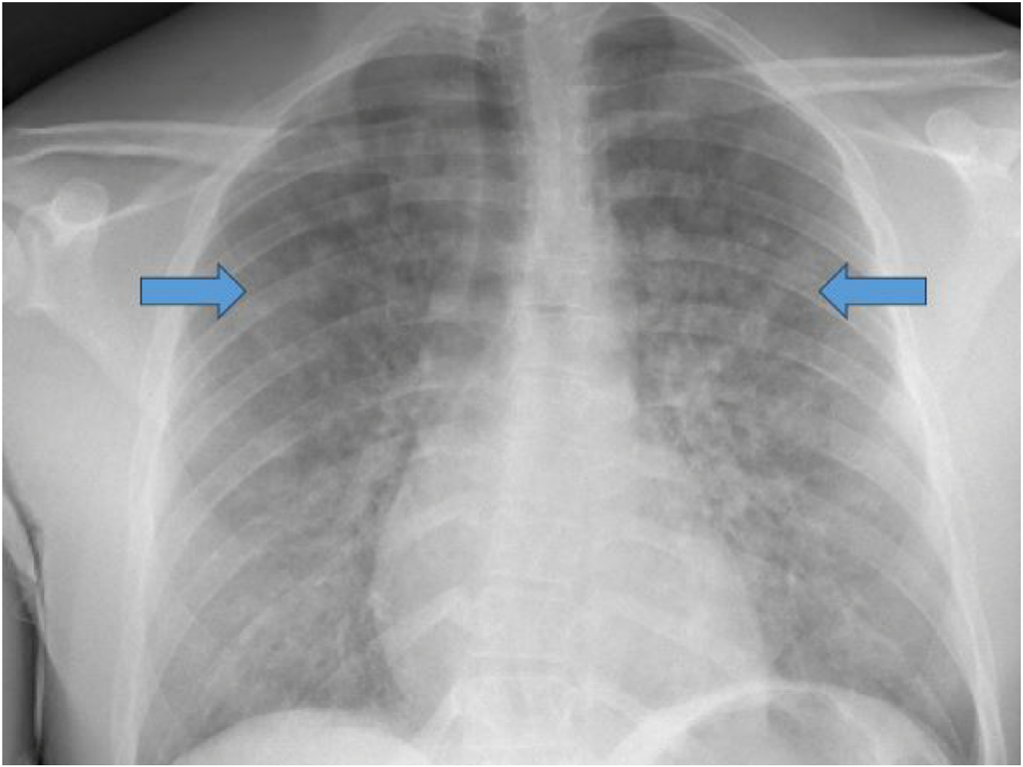
Upright anterior-posterior chest radiograph in a patient with diffuse alveolar hemorrhage, demonstrating patchy alveolar opacities (arrows).

**Image 2.**
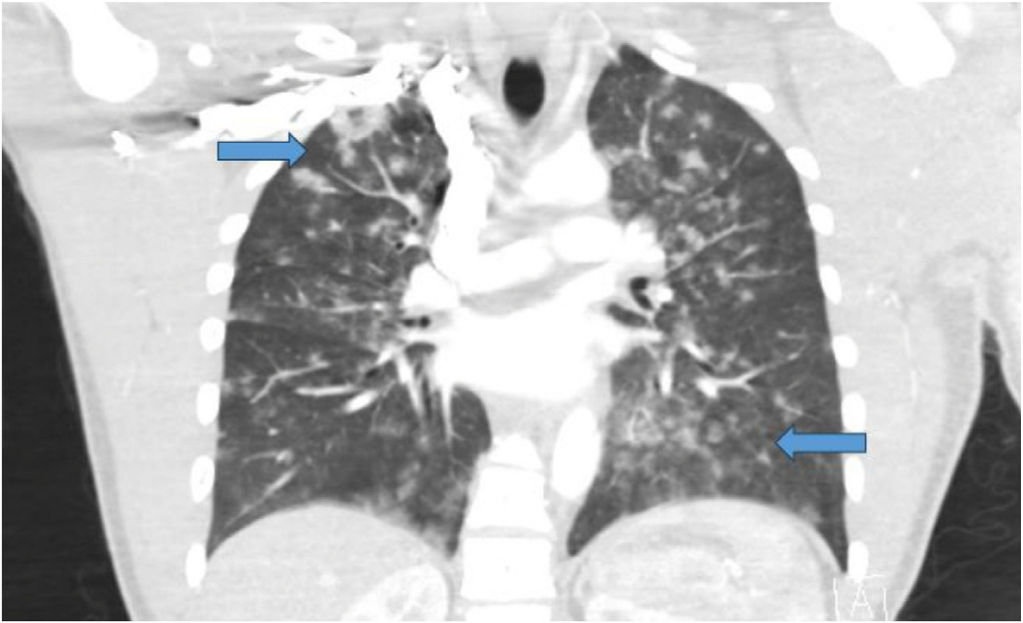
Coronal view of computed tomography angiogram of the chest demonstrating diffuse alveolar hemorrhage (arrows).

On arrival to the ED, the patient was found to be tachypneic with a respiratory rate of 24 breaths per minute. The PACU staff had reported hypoxemia to the EMS personnel, and he was transported on four liters of oxygen via nasal cannula with an ED oxygen saturation of 96%. He was normotensive without tachycardia. The patient had hemoptysis in the ED with bilateral rales on auscultation of his lungs. A portable chest radiograph (Image 1) was obtained, which showed diffuse multifocal patchy alveolar opacities in the bilateral lungs concerning for DAH. Chest computed tomography angiogram (Image 2) further supported the diagnosis of DAH, without any findings of pulmonary embolism, pneumothorax, or other significant chest pathology. Initial laboratory work-up revealed a lactic acid level of 6.8 millimoles per liter (mmol/L) (reference range 0.5–2.2 mmol/L), a hemoglobin level of 14.8 grams per deciliter (g/dL) (14.0–18.0 g/dL), a platelet count of 227,000 per microliter (μL) (150,000–450,000/μL), an international normalized ratio of 0.99 (0.85–1.17), a partial thromboplastin time of 23.2 seconds (22.9–35.8 seconds), and a creatinine level of 1.49 milligrams (mg) per dL (0.50–1.40 mg/dL).

During the patient’s ED stay, he was administered one liter of intravenous crystalloid solution for his lactic acidosis and acute kidney injury. His tachypnea and hemoptysis improved, and he was gradually weaned from supplemental oxygen. After pulmonology consult in the ED, the patient was admitted to inpatient medicine for overnight observation and further work-up of his hemoptysis. He underwent bronchoscopy with bronchoalveolar lavage the next day with results consistent with DAH. He also received lab work-up for autoimmune processes including antinuclear antibody, complement component 3, complement component 4, rheumatoid factor, anti-double stranded DNA antibody, and anti-neutrophil cytoplasmic antibody, all of which were within normal limits. The patient remained clinically stable and experienced resolution of his lactic acidosis (1.3 mmol/L) and acute kidney injury (creatinine 1.10 mg/dL) and was discharged the day after admission with outpatient follow-up.

## DISCUSSION

Diffuse alveolar hemorrhage is a rare but serious complication during the perioperative period. One mechanism that has been proposed to cause DAH during the perioperative period is laryngospasm. Laryngospasm is the sustained closure of the vocal cords that results in the loss of a patent airway; its incidence during anesthesia has been reported to be just under 1%.^1^ Patients who undergo endotracheal intubation are most likely to experience laryngospasm after extubation during the emergence period, likely due to decreased conscious control of the laryngeal closure reflex.^2^ Factors associated with higher incidence of laryngospasm include use of volatile, inhaled anesthetics, younger patients, reactive airway disease, smoking, and airway procedures. Young athletic males are the subjects in most case reports and case series regarding episodes of alveolar hemorrhage and edema related to anesthesia.^3^ ^–^ ^8^ It is hypothesized that when experiencing episodes of laryngospasm, young athletic males generate significant negative inspiratory force that can cause injury to the tracheobronchial vasculature.

Another mechanism that has been proposed as a cause of perioperative DAH is the use of the inhaled anesthetic sevoflurane. Sevoflurane is a volatile, halogenated gas used as an inhaled anesthetic for induction and maintenance of anesthesia. Well known side effects of volatile anesthetics include nausea, vomiting, hypotension, arrhythmias, coughing, breath-holding, respiratory depression, and laryngospasm. Case reports describing episodes of pulmonary alveolar hemorrhage after sevoflurane use are sparse and generally only described in the anesthesia literature. These case reports describe patients who were young healthy males undergoing routine surgical procedures with general anesthesia.^3^ ^,^ ^9^ ^–^ ^12^ Volatile anesthetics are known to be lipid soluble and activate the arachidonic cascade within the cell membrane, which may increase alveolar permeability and oxidative stress.^13^ Sevoflurane specifically has been shown in vitro to inhibit platelet aggregation.^14^ These properties may increase the risk of DAH in patients who are susceptible.

## CONCLUSION

This case supports previously published reports of sevoflurane-induced diffuse alveolar hemorrhage. Interestingly, other than naloxone-mediated acute flash pulmonary edema, there are no reports of any intravenous anesthetics or reversal agents resulting in DAH. Hence, the most likely causative agent in this case was the volatile, inhaled anesthetic sevoflurane, as there was no report of laryngospasm at the time of extubation. This represents the first reported case to our knowledge in the EM literature, likely due to the rarity of this diagnosis and the fact that most cases occur in the PACU. With outpatient surgical centers becoming an increasingly popular option for lower risk procedures, emergency physicians would benefit from understanding this presentation and its pathophysiology.
